# Supplementary material for: Gluten-Free Sorghum Pasta: Composition and Sensory Evaluation with Different Sorghum Hybrids
Source: Foods. 2022 Oct 8;11(19):3124. doi: 10.3390/foods11193124 (PMC9563424; doi:10.3390/foods11193124)
Supplement: Supplementary file 1 [file foods-11-03124-s001.zip › foods-1912970-supplementary.pdf]

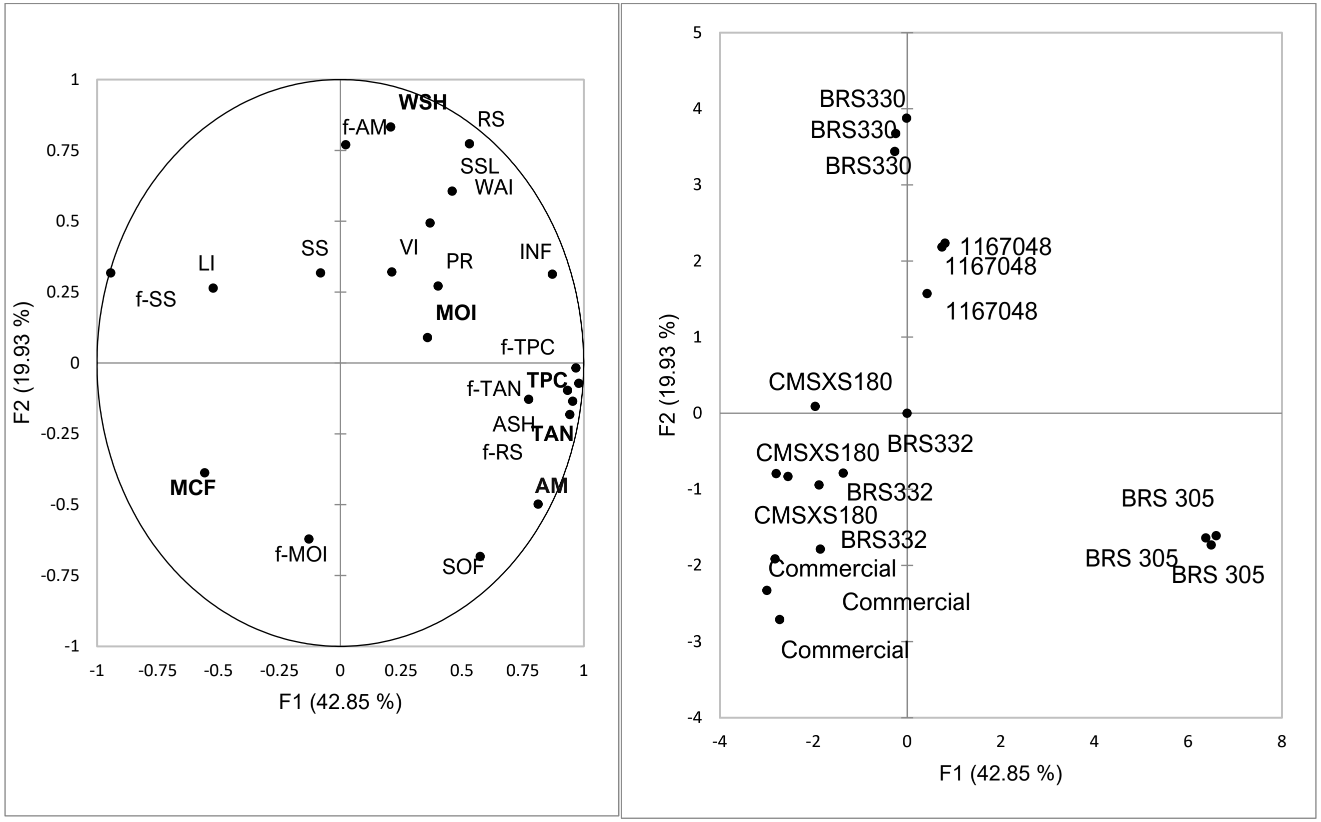

Supplementary electronic Figure S1. PCA of chemical characteristics of sorghum flours and the physical, chemical and technological characteristics of their respective cooked pastas. Variables of cooked pastas are in bold. Variables are: PR - protein content; LI - lipid content; SOF – soluble fibers; INF – insoluble fibers; ASH - ashes; TPC - total phenolic contents; TAN - total condensed tannins; DPPH- antioxidant activity EC50; FRAP - antioxidant activity; RS - resistant starch; f-RS: resistant starch in the flour; AM - amylose content; f- AM: Amylose in the flour; SS - soluble starch; f- SS – Soluble starch in the flour; MOI - moisture; f- MOI: moisture in the flour; WSH - work of shear; MCF - maximum cutting force; WAI - water absorption index; VI - volume increase; SSL- soluble solids losses.
